# Homogenization of Through-Thickness Microstructure and Mechanical Properties in Direct-Quenched High-Nb Q690 Steel via Tempering

**DOI:** 10.3390/ma19040792

**Published:** 2026-02-18

**Authors:** Haonan Wang, Dazhao Li, Yongqing Zhang, Peimao Fu, Haitao Lu, Hejia Zhu, Xingchi Chen, Boyu Guan, Yongan Chen, Shaobin Bai

**Affiliations:** 1 School of Materials Science and Engineering, North University of China, Taiyuan 030051, China; 13467164352@163.com (H.W.);; 2 Shanxi Key Laboratory of Advanced Metal Materials for Special Environments, Taiyuan 030051, China; 3 CITIC-CBMM Microalloying Technology Center, CITIC Metal Co., Ltd., Beijing 100004, China; zhangyq@citic.com; 4 Technology Center, Shanxi Taigang Stainless Steel Ltd., Taiyuan 030003, China; sxtyfupm@163.com; 5 School of Materials Science and Engineering, Taiyuan University of Science and Technology, Taiyuan 030024, China; b202314110015@stu.tyust.edu.cn; 6 School of Semiconductor and Physics, North University of China, Taiyuan 030051, China; 7 School of Aerospace Engineering, North University of China, Taiyuan 030051, China

**Keywords:** high-Nb steel, direct quenching and tempering, M-A constituent, through-thickness homogeneity, strengthening mechanism

## Abstract

Manufacturing heavy-gauge high-strength steel plates with uniform through-thickness properties is challenging due to the limited hardenability and significant cooling rate variations inherent to heavy sections. However, the mechanism governing microstructural homogenization across such large cross-sections remains not fully understood. This study investigates the through-thickness microstructure and mechanical properties of a 60 mm thick high-Nb microalloyed Q690 steel plate processed by direct quenching (AQ) and subsequent tempering at 530 °C and 580 °C. Characterization was performed at the surface (0t), quarter-thickness (1/4t), and core (1/2t) locations. Results revealed a pronounced gradient in the as-quenched state: while the surface consisted of fine lath martensite/bainite, the core formed coarse granular bainite containing blocky martensite–austenite (M-A) constituents. This microstructural heterogeneity resulted in poor core toughness (~24 J). High-temperature tempering at 580 °C promoted the complete decomposition of these metastable M-A constituents into ferrite and fine carbides, significantly improving the core impact energy to ~49 J. However, a toughness gradient persisted compared to the quarter-thickness (>120 J), attributed to the inherited coarse matrix and the formation of grain boundary carbides. Notably, high yield strength was maintained across the thickness despite matrix recovery. This is primarily attributed to a potent anti-softening effect provided by thermally stable (Nb,Ti,Mo)C nanoprecipitates, which generate strong Orowan strengthening. These findings highlight the critical role of optimizing the trade-off between M-A decomposition and carbide evolution in promoting the microstructural and property homogenization of heavy-gauge steels.

## 1. Introduction

In recent years, the trend towards the use of larger-scale and lightweight equipment in sectors such as coal mining, engineering machinery, and marine engineering has imposed increasingly stringent demands on the strength, toughness, and weldability of structural steels [1,2,3]. As a typical representative of high-strength low-alloy (HSLA) steels, Q690 steel is widely used in critical load-bearing components of engineering machinery and coal mining machinery due to its excellent combination of strength and toughness. However, manufacturing heavy-gauge (≥60 mm) high-strength steel plates with uniform through-thickness properties poses a significant challenge for the steel industry, especially given their increasingly harsh service environments [4,5].

In heavy-gauge steel plates, the ‘thickness effect’ constitutes a primary obstacle to performance enhancement [6,7,8]. During heat treatment, a significant gradient in quenching severity (i.e., cooling rate) arises from the surface to the core. Fundamentally, the cooling rate governs phase transformation kinetics and the resulting microstructural heterogeneity, as evidenced by continuous cooling transformation (CCT) studies [9]. Consequently, high quenching severity at the surface suppresses atomic diffusion, promoting a shear-dominated transformation that results in hard lath martensite or bainite with high dislocation density. Conversely, the diminished cooling rate at the core promotes the decomposition of undercooled austenite through diffusional or semi-diffusional transformations. This leads to the formation of coarse granular bainite (GB) and proeutectoid ferrite, along with the retention of coarse martensite–austenite (M-A) constituents. This microstructural heterogeneity through the thickness inevitably leads to inconsistent mechanical properties, most notably a drastic deterioration in the impact toughness of the core region. Consequently, the structural integrity and in-service safety of components are severely compromised. Therefore, achieving through-thickness property homogeneity by refining the core microstructure via composition and process optimization remains a critical technical challenge.

In steel plate manufacturing, Direct Quenching (DQ) has gained prominence as an alternative to the conventional offline Reheating and Quenching (RQ) process, owing to its advantages in energy savings, shorter production cycles, and improved operational efficiency [10,11]. The DQ process utilizes the residual heat from hot rolling for immediate quenching. This approach preserves the deformation-strengthened austenite structure, leading to a refined final microstructure [12]. However, compared to the RQ process, DQ exhibits higher sensitivity to rolling parameters and the subsequent cooling path [13]. Furthermore, in the production of heavy-gauge plates, solely relying on the adjustment of processing parameters often proves insufficient to completely eliminate microstructural coarsening induced by the limited cooling rate at the core.

To overcome these processing limitations, microalloying has been extensively employed in the development of high-strength steels [4,14,15,16,17,18,19]. Niobium (Nb) is a particularly effective microalloying element due to its dual role in grain refinement and precipitation strengthening [20]. The high strength of such microalloyed steels is derived from a synergy of mechanisms: grain boundary strengthening via the Hall–Petch relationship, precipitation strengthening from nanoscale carbides pinning dislocations (Orowan mechanism), and substructural strengthening from the high dislocation density preserved by the DQ process. In conventional microalloyed steels, Nb content is typically kept below approximately 0.05 wt.%. However, recent studies suggest that a ‘high-Nb’ strategy, characterized by increased Nb content, can be highly beneficial [21,22]. The solute drag effect of Nb in austenite significantly retards recrystallization, leading to a refined prior austenite grain size [23]. Furthermore, during tempering, the precipitation of Nb-rich carbides provides substantial additional strengthening [22,24]. This approach thus presents a promising route to improve the core microstructure and enhance the strength–toughness balance of heavy-gauge plates.

While both high-Nb microalloyed steels and the DQ process have been extensively studied separately, systematic research on the through-thickness microstructural evolution in heavy-gauge Q690 plates processed by the combined ‘DQ + tempering’ route is scarce. In particular, the tempering-induced decomposition of metastable core phases, especially M-A constituents, and its consequent effect on the strengthening and toughening mechanisms remain poorly understood. To address this gap, the present work investigates a 60 mm thick high-Nb microalloyed Q690 steel plate processed by industrial direct quenching and simulated tempering. The primary objective of this study is to systematically characterize the through-thickness microstructural heterogeneity in the as-quenched plate, thereby identifying the microstructural origins of the toughness gradient. Furthermore, we aim to investigate the evolution of metastable phases during high-temperature tempering, with a specific focus on the decomposition behavior of the core M-A constituents. Finally, this work seeks to elucidate the synergistic strengthening and toughening mechanisms, determining how the interaction between thermally stable nanoprecipitates and matrix recovery governs the overall mechanical properties. The findings are expected to provide a theoretical foundation for optimizing the heat treatment of heavy-gauge engineering machinery steels.

## 2. Materials and Methods

The material used in this study was a high-Nb microalloyed Q690 steel manufactured via an industrial process. The chemical composition, including both nominal design and actual values determined by spark optical emission spectrometry (Spark-OES, ARL 4460, Thermo Fisher Scientific, Ecublens, Switzerland), is presented in Table 1. The steel was melted in an electric arc furnace (EAF), followed by ladle furnace (LF) refining and vacuum degassing (VD) to ensure compositional homogeneity and low impurity levels. The steel was then cast and reheated to 1200 °C for 150 min to homogenize the austenite and dissolve microalloying elements. Subsequently, it was rolled into 60 mm thick plates on an industrial heavy plate rolling line. Immediately after the final rolling pass, the plates were subjected to an industrial on-line DQ process. The quenching start temperature (QST) was controlled at about 900 °C and water spray cooling from the production line’s accelerated cooling system was applied until the quenching finish temperature (QFT) fell below 150 °C. The water spray pressure was maintained in the range of 0.3–0.5 MPa to ensure sufficient droplet impact velocity. Although some localized vapor phase effects may persist compared to polymer immersion, the high kinetic energy of the spray droplets destabilizes the stable vapor film (Leidenfrost effect) [25], facilitating a transition to nucleate boiling. While the core cooling rate of the 60 mm thick plate is ultimately conduction-limited, this method maximizes surface heat extraction to maintain the steepest possible thermal gradient.

**Figure 1:**
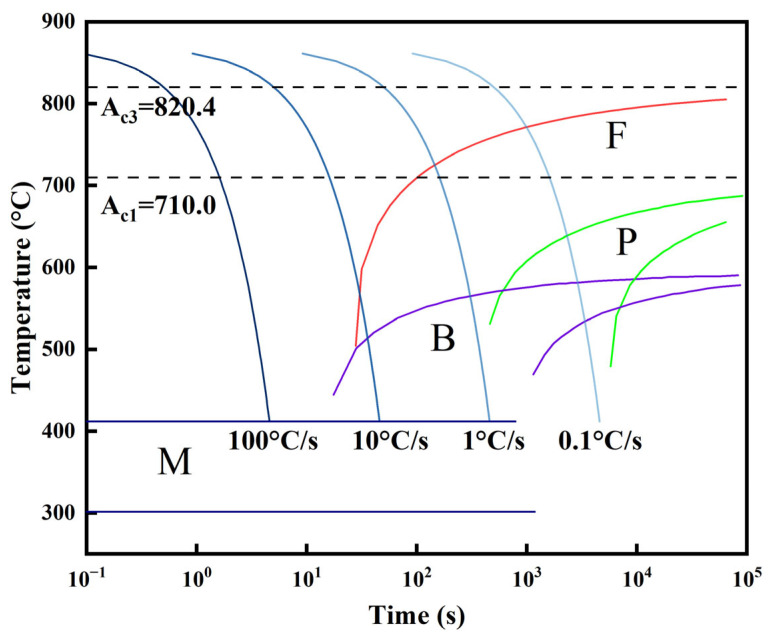
CCT diagram of the experimental steel. The red, green, purple, and blue curves represent the ferrite (F), pearlite (P), bainite (B), and martensite (M) transformation regions, respectively. The black dashed lines indicate the critical temperatures Ac1 and Ac3.

After the industrial production of the as-quenched plates was completed, test blocks were extracted from the plates for subsequent heat treatment. These specimens were tempered offline in a laboratory box-type resistance furnace at 530 °C and 580 °C for 1.5 h, followed by water cooling to room temperature to suppress temper embrittlement [26]. These conditions are designated as ‘AQ’ (as-quenched), ‘T530’, and ‘T580’, respectively. Additionally, the Ac3 and Ac1 transformation temperatures were calculated using JMatPro v7.0 software to be 820.4 °C and 710.0 °C, respectively. The corresponding simulated CCT curves are presented in Figure 1. The simulation was conducted using the ‘General Steel’ module with a prior austenite grain size of ASTM 9.

**Figure 2:**
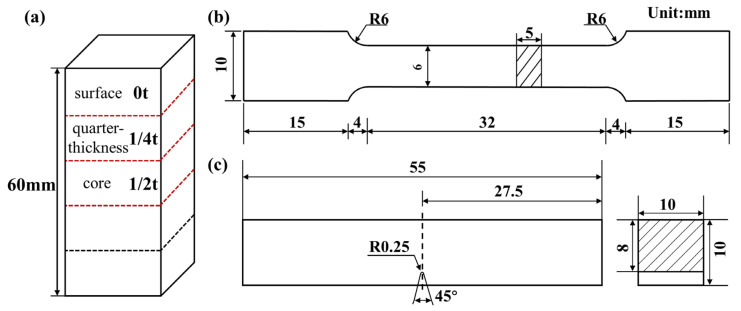
Schematic diagrams of (**a**) sampling positions along the thickness direction, and geometries of (**b**) the flat tensile specimen and (**c**) the Charpy V-notch impact specimen.

To assess the through-thickness uniformity of mechanical properties after different heat treatments, specimens were taken from the upper surface (designated 0t), quarter-thickness (1/4t), and core (1/2t) positions of the AQ, T530 and T580 plates, as illustrated in Figure 2a. Specimens for metallography, flat tensile testing (geometry detailed in Figure 2b), and standard Charpy V-notch impact testing (Figure 2c) were all sectioned along the rolling direction using wire electrical discharge machining (WEDM, Taizhou Ruilin CNC Machine Tool Co., Ltd., DK7745, Taizhou, China). Room-temperature tensile tests were performed on an AG-X PLUS universal testing machine (Shimadzu Corporation, Kyoto, Japan) at a strain rate of 0.001 s^−1^. Charpy impact tests were conducted at 20 °C on a JB-W300DY pendulum impact tester (Shandong Shijin Testing Machine Co., Ltd., Qingdao, China). Vickers hardness (HV1) was measured using a JMHVS-1000AT tester (Shanghai Aolong Xingdi Testing Equipment Co., Ltd., Shanghai, China) with a 1 kgf load and a 10 s dwell time. Each reported value is the average of five independent measurements.

After mechanical grinding and polishing, the specimens were etched with a 4% nital solution. The microstructural characterization was performed using a SIGMA500 field emission scanning electron microscope (SEM, ZEISS, Oberkochen, Germany). Phase analysis was conducted using a Rigaku SmartLab X-ray diffractometer (XRD, Rigaku Corporation, Tokyo, Japan) equipped with a Cu Kα radiation source, operating at 40 kV and 30 mA. Scans were performed over a 2θ range of 30° to 90° at a speed of 2°/min. To further characterize the morphology and structure of nanoscale precipitates, a Tecnai G2 F30 transmission electron microscope (TEM, FEI Company, Hillsboro, OR, USA) equipped with energy-dispersive X-ray spectroscopy (EDS) was employed.

## 3. Results

### 3.1. Distribution of Mechanical Properties Along the Thickness Direction

**Figure 3:**
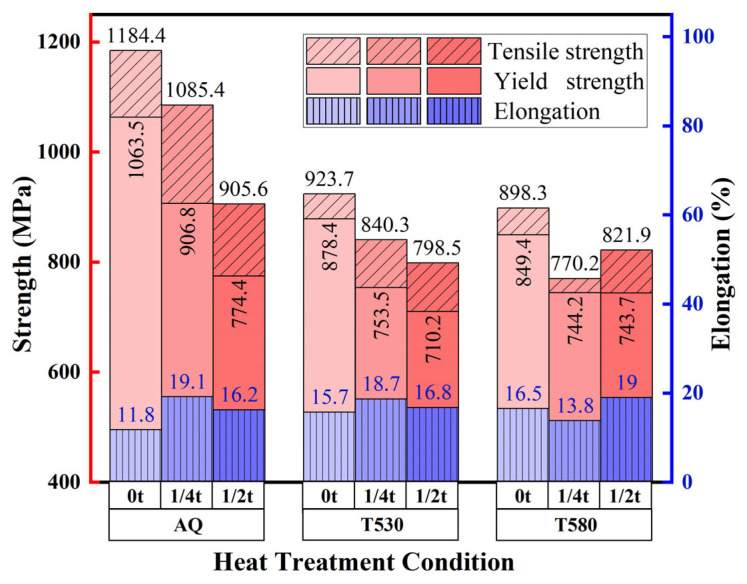
Through-thickness variations in tensile properties for the AQ, T530 and T580 specimens.

Figure 3 presents the tensile properties of the high-Nb steel across the thickness in the AQ, T530, and T580 conditions. A consistent strength gradient was observed under all conditions, with strength decreasing from the surface to the core. The AQ condition showed the highest overall yield strength (YS) but also the most pronounced strength gradient. The YS at the surface reached 1063 MPa, significantly exceeding the standard Q690 requirement. This ultra-high strength is primarily attributed to the synergistic strengthening from the refined lath martensite substructure, high dislocation density, and precipitation of fine carbides. Correspondingly, the YS decreased progressively to 774 MPa at the core.

Tempering effectively mitigated this through-thickness strength decline. For T530, the surface YS decreased to 878 MPa. Although the core strength was lower (710 MPa), it was close to the value at the 1/4t position (753 MPa). T580 resulted in a further reduction in surface strength (849 MPa) and led to nearly identical strengths at 1/4t (744 MPa) and the core (743 MPa). These results demonstrate that tempering, particularly at higher temperatures, significantly improves the through-thickness homogeneity of strength.

**Figure 4:**
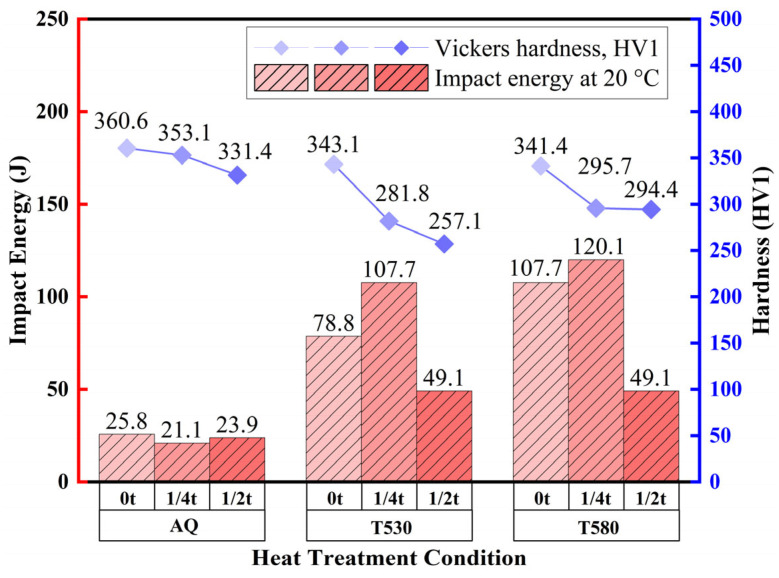
Through-thickness distributions of room-temperature impact toughness and Vickers hardness for the AQ, T530 and T580 specimens.

Figure 4 presents the room-temperature impact toughness and Vickers hardness distribution across the thickness for the as-quenched and tempered conditions. In the AQ state, despite the variation in strength, the impact energy remained uniformly low (~20 J) at all positions, highlighting the inherent brittleness of the as-quenched microstructure.

Tempering markedly improved the impact toughness. This improvement was most pronounced in T580, where the impact energy reached 107 J at the surface and 120 J at the 1/4t position. Even the core exhibited a significantly higher value of 49 J compared to the as-quenched state. A similar, though less pronounced, improvement was observed in T530. These results indicate that tempering, especially at higher temperatures, effectively improves toughness by modifying the brittle microstructural constituents formed during quenching.

In the AQ state, the hardness distribution exhibited a typical decreasing gradient along the thickness direction [6]. The surface exhibited the highest hardness of 360.6 HV, followed closely by 353.1 HV at the quarter-thickness position, while the core showed the lowest value of 331.4 HV. This gradient is primarily attributed to the variation in cooling rates across the thickness: the rapid cooling rate at the surface promoted the formation of hard, fine lath martensite, whereas the slower cooling rate at the core led to the formation of softer, coarse granular bainite, resulting in reduced hardness.

Upon tempering, the hardness distribution tended towards homogenization. This effect was most pronounced in T580, where the hardness variation across the layers was minimized, and the values at the quarter-thickness and core positions became nearly consistent. This indicates that high-temperature tempering not only relieved quenching stresses but also effectively narrowed the property gradient caused by microstructural heterogeneity.

### 3.2. Heterogeneous Microstructures Along the Thickness Direction

**Figure 5:**
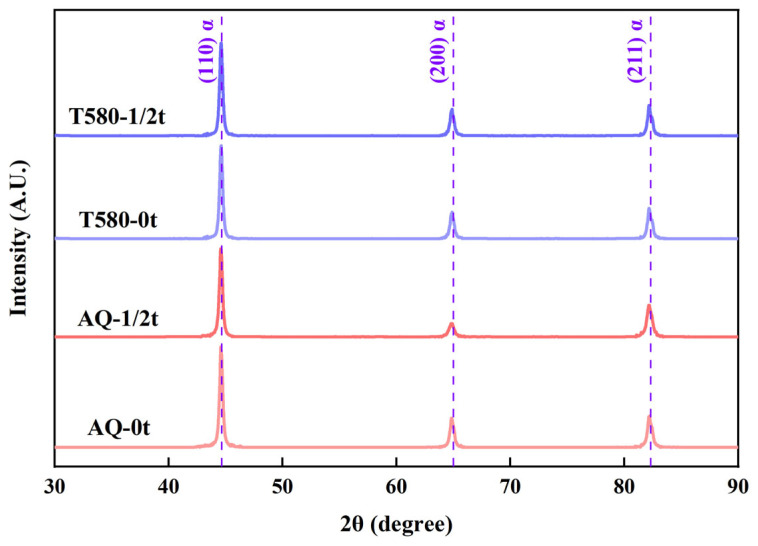
XRD patterns of the surface and core specimens in the AQ and T580 specimens.

To elucidate the microstructural origin of the mechanical property gradients, XRD analysis was performed on the surface and core specimens of the AQ and T580 states. The obtained XRD patterns are presented in Figure 5. Notably, no distinct diffraction peaks corresponding to retained austenite were observed in either the AQ or T580 conditions, indicating that its volume fraction is below the detection limit of the XRD analysis.

**Figure 6:**
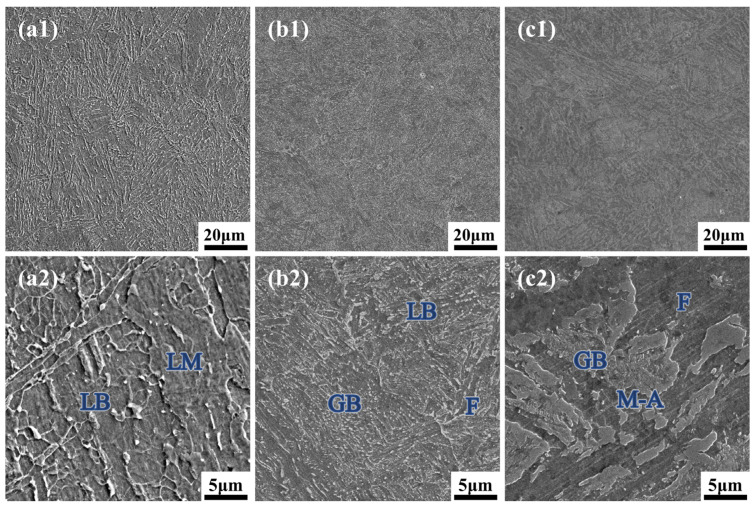
SEM micrographs of the AQ specimen along the thickness direction. (**a1**,**a2**) 0t, characterized by a mixture of LM and LB; (**b1**,**b2**) 1/4t, dominated by lath bainite and GB with fine M-A constituents; and (**c1**,**c2**) 1/2t, consisting of coarse granular bainite with large, blocky M-A constituents.

The through-thickness microstructures of the AQ and tempered specimens were characterized. Figure 6 shows the SEM micrographs of the AQ specimen. Quantitative analysis using the linear intercept method indicates that the prior austenite grain size (PAGS) ranges from 15 to 22 μm (ASTM 8–9), which is consistent with the initial setting (ASTM 9) used in the JMatPro simulation. Its microstructure, resulting from direct quenching, consists of lath martensite (LM), lath bainite (LB), granular bainite (GB), ferrite (F), and M-A constituents. Owing to the decreasing cooling rate from surface to core, a pronounced microstructural gradient is evident.

At the 0t, the microstructure is primarily a mixture of lath bainite and lath martensite. The bainite forms coarse, closely packed lath packets, with discontinuous chain-like carbides at the interfaces. The martensite appears as blocky packets that exhibit strong surface relief, indicative of its high dislocation density, which contributes to the high strength of this region. At 1/4t, the microstructure is dominated by lath bainite and granular bainite. The lath bainite is finer and more directionally aligned compared to the surface. The granular bainite consists of fine, bright-etching M-A constituents embedded in an irregular ferrite matrix. The 1/2t, experiencing the slowest cooling, transforms into coarser granular bainite. Here, the granular bainite features particularly large M-A constituents. Quantitative analysis revealed a high volume fraction of 19.4% M-A constituents with a heterogeneous size distribution (0.75 ± 0.47 µm), characterized by the presence of coarse blocky islands. The significant hardness mismatch between this soft matrix and the large, hard M-A constituents acts as a primary driver for stress concentration and potential microcrack initiation [27].

**Figure 7:**
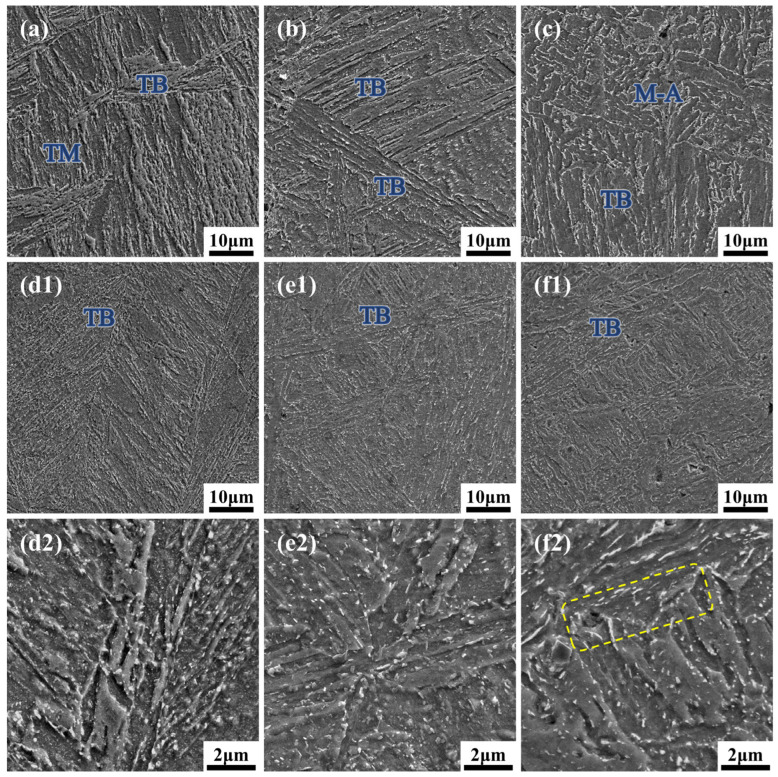
SEM micrographs of the tempered specimens along the thickness direction. (**a**–**c**) T530 condition: (**a**) 0t consisting of TB and TM; (**b**) 1/4t exhibiting coarsened bainitic structures; (**c**) 1/2t exhibiting residual, incompletely decomposed M-A constituents. (**d**–**f**) T580 condition: (**d1**,**d2**) 0t characterized by well-recovered feather-like tempered bainite; (**e1**,**e2**) 1/4t with uniformly dispersed granular carbides; (**f1**,**f2**) 1/2t showing complete decomposition of M-A constituents into fine carbides and ferrite matrix. Yellow boxes indicate the chain-like carbides distributed along grain boundaries.

To mitigate the microstructural and property gradients present in the AQ state, tempering heat treatment was performed. Figure 7 illustrates the microstructural evolution after tempering at 530 °C and 580 °C, revealing a significant trend toward homogenization.

For T530, the microstructure transformed into a mixture consisting primarily of tempered bainite (TB) and tempered martensite (TM). In the surface layer, although the bainitic lath boundaries became blurred, the original orientation relationship was retained, and the lath interiors underwent significant recovery, leading to a reduction in dislocation density. Simultaneously, the lath martensite decomposed, precipitating granular carbides dispersed throughout the ferrite matrix. At the 1/4t position, both lath-like and granular bainite exhibited signs of coarsening. Notably, the decomposition of the coarse M-A constituents in the core was incomplete. Although the volume fraction significantly decreased to 3.1% and the average size was refined to 0.36 ± 0.11 µm, indicating partial decomposition, residual island-like M-A structures persisted. This incomplete phase transformation explains why the through-thickness property gradient, while mitigated, remained evident in the T530 condition.

In contrast, tempering at 580 °C enhanced atomic diffusion and microstructural recovery. In the surface layer, significant dislocation recovery within the laths facilitated the formation of typical feather-like tempered bainite. The 1/4t region exhibited a uniform dispersion of granular carbides. Most critically, the brittle M-A constituents in the core decomposed completely (becoming negligible in quantitative analysis), transforming into fine carbides distributed within the ferrite matrix. This thorough through-thickness microstructural homogenization—specifically the elimination of blocky M-A constituents containing high-carbon martensite—directly elucidates the microstructural origin of the excellent and uniform impact toughness achieved in the T580 state.

### 3.3. Analysis of Nanoscale Precipitates

**Figure 8:**
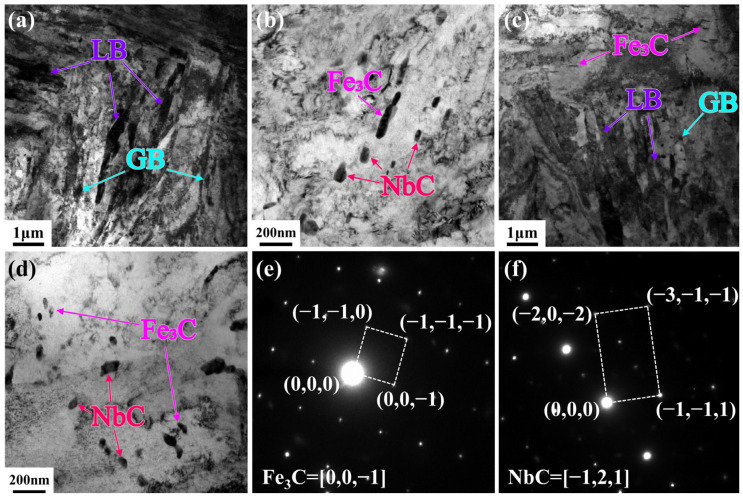
TEM bright-field images and SAED patterns of the T580 specimen. (**a**,**b**) Bright-field images of the 0t and (**c**,**d**) 1/2t, revealing the dispersion of precipitates within the ferrite matrix; (**e**) SAED pattern identifying the short-rod or granular cementite Fe_3_C; (**f**) SAED pattern identifying the cube-shaped Nb-rich carbides NbC.

To elucidate the mechanism underlying the retained high strength after high-temperature tempering, the T580 specimen was characterized in detail using TEM. Figure 8a presents a TEM bright-field image revealing two distinct populations of precipitates within the ferrite matrix. The first type consists of cementite particles exhibiting short-rod or granular morphologies, with sizes ranging from tens to several hundred nanometers. The second type, which is inferred to be the primary contributor to precipitation strengthening, is identified as cube-shaped Nb-rich carbides (NbC) with a size of approximately tens of nanometers.

**Figure 9:**
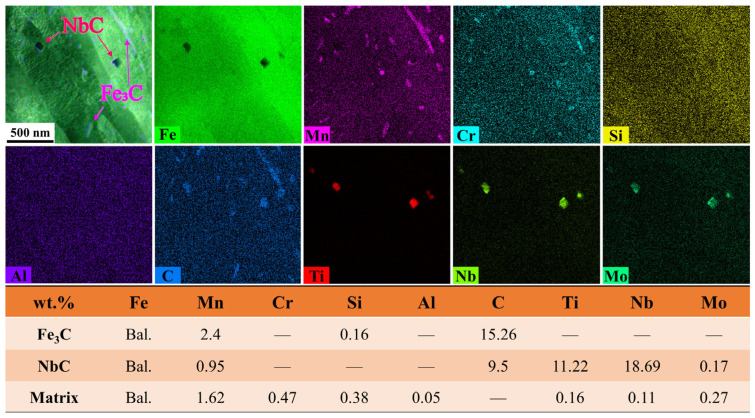
EDS elemental analysis of the cementite and Nb-rich carbides in the T580 specimen, revealing Mn/Cr partitioning in cementite ((Fe,Mn,Cr)_3_C) and Nb/Ti/Mo enrichment in cubic carbides ((Nb,Ti,Mo)C). The symbol “—” indicates that the element was not detected.

EDS analysis (Figure 9) confirms that Mn and Cr partitioned into the cementite, substituting for Fe to form alloyed (Fe,Mn,Cr)_3_C. This elemental enrichment enhances the thermal stability of the cementite, effectively retarding its coarsening during tempering. In contrast, the cubic precipitates are enriched in Nb, Ti, and Mo, identifying them as complex (Nb,Ti,Mo)C carbides. Their formation is primarily driven by the strong chemical affinity of Nb and Ti for carbon [24]. Crucially, the incorporation of Mo into the NbC lattice reduces the lattice misfit between the carbide and the ferrite matrix. This reduction in lattice misfit lowers the interfacial energy of the system, thereby stabilizing the precipitates and suppressing their growth [28]. This mechanism promotes the formation of finer, high-density carbides, which are essential for the retained high strength.

These fine, stable (Nb,Ti,Mo)C precipitates effectively pin dislocations through the Orowan mechanism, providing strong precipitation strengthening [29]. Research on the kinetics and strengthening mechanisms of Nb-bearing high-strength steel confirmed that nanoscale Nb-based carbides contribute the primary strength increment through the Orowan bypassing mechanism, serving as a decisive factor for yield strength [16]. Furthermore, this strengthening effect aligns with the observations of Pan et al., who indicated that precipitates effectively compensate for the strength loss induced by matrix recovery during tempering by impeding dislocation motion [30]. Consequently, the synergy between maintaining high strength via precipitation and improving toughness through matrix recovery constitutes the fundamental microstructural basis for the excellent strength–toughness balance achieved in this steel.

## 4. Discussion

### 4.1. Effect of Cooling Rate and Hardenability on Microstructural Evolution

The pronounced microstructural and property gradients in the as-quenched steel fundamentally stem from the through-thickness cooling rate variation during DQ. This evolution can be elucidated by correlating the microstructures with the simulated CCT curves (Figure 1). Based on the plate thickness and industrial quenching parameters, the cooling rates are estimated to vary significantly, ranging from approximately >30 °C/s at the surface to 4 °C/s at the core [31].

Consequently, the 0t and 1/4t regions, experiencing relatively fast cooling, bypassed the high-temperature ferrite and pearlite transformation zones. At the surface, the high cooling rate restricted atomic diffusion, promoting a shear-dominated transformation that produced a mixture of lath bainite and lath martensite with a high dislocation density. Due to the significant plate thickness, residual heat conducted from the core induced an “auto-tempering” effect on this surface microstructure. This process facilitated partial lath recovery and coalescence and promoted the precipitation of fine carbides within the martensitic laths, as observed in the SEM analysis. At the 1/4t position, the slightly reduced cooling rate raised the local transformation start temperature, enhancing carbon diffusion. During lath bainite growth, carbon partitioned into the surrounding austenite, reducing its solute drag on interfaces. This enabled interfacial migration and lath coalescence, leading to the formation of granular bainite alongside lath bainite.

In contrast, the core region experienced a drastically reduced cooling rate (estimated at 4 °C/s) due to significant thermal lag. This cooling trajectory entered the relatively high-temperature bainitic transformation domain on the CCT diagram, where transformation exhibited mixed shear and diffusion characteristics. The higher transformation temperature enabled sufficient carbon diffusion. Carbon was rejected from the proeutectoid or bainitic ferrite and enriched at the interfaces of untransformed austenite, fostering coarse granular bainite, where the reduced cooling rate promotes carbon enrichment in the residual austenite [32], chemically stabilizing it against martensitic transformation. The carbon-enriched austenite islands were stabilized and retained during primary cooling. Upon further cooling, inhomogeneous carbon distribution within these islands led to a localized martensitic transformation in lower-carbon regions below their Ms temperature, while higher-carbon cores potentially remained as retained austenite. This process resulted in coarse, blocky M-A constituents, which are primarily responsible for the poor toughness at the core [33,34]. It is worth noting that despite the low alloy content, the synergistic addition of Mn, Cr, and Mo effectively retards the diffusional transformation kinetics of polygonal ferrite and pearlite [35,36]. This enhanced hardenability was crucial for maintaining a bainite-dominated matrix even at the core, preventing the formation of equilibrium phases under the reduced cooling rate.

### 4.2. Role of Tempering Temperature in Balancing Strength and Toughness

While the as-quenched state offers high strength, its low toughness necessitates tempering to achieve a balanced property profile. This process relies on thermal activation to drive carbon diffusion and defect annihilation. Compared to tempering at 530 °C, the 580 °C treatment provides greater thermodynamic driving force, playing a decisive role in restoring through-thickness toughness by decomposing metastable phases and promoting matrix recovery. The decomposition of M-A constituents is a primary factor in toughness recovery. In the as-quenched state, these hard, brittle islands act as stress concentrators and preferential sites for cleavage crack initiation under impact [34]. Tempering decomposes them into a softer ferrite matrix and carbides, eliminating these critical crack nuclei. The 580 °C treatment ensures their complete decomposition, yielding fine, spherical carbides uniformly dispersed in the ferrite or at sub-boundaries (Figure 7). However, the toughening effect is significantly influenced by the inherited microstructure from quenching. At the 1/4t, the initial lath bainite mainly undergoes polygonization during tempering, retaining a fine structure. The synergy of this refined microstructure and the elimination of brittle phases makes crack initiation difficult and propagation paths tortuous, leading to a dramatic increase in impact energy from ~21 J to over 120 J.

In contrast, the core exhibits a more limited toughness improvement (from ~24 J to ~49 J), which is comparable to the value obtained after 530 °C tempering. This phenomenon can be explained by a competitive mechanism involving microstructural evolution. While the higher temperature (580 °C) facilitates the fuller decomposition of brittle M-A constituents, the higher carbon content and slower cooling rate in the core also promote the coarsening and aggregation of carbides along prior austenite or ferrite grain boundaries. Unlike the fine dispersed carbides at the surface, these coarsened, chain-like grain boundary carbides readily cause stress concentration and facilitate intergranular crack propagation. This failure mechanism aligns with fractographic findings in similar bainitic HSLA steels [37], which identified such coarse phases as preferential initiation sites for cleavage cracks. Consequently, the detrimental effect of carbide coarsening partially offsets the toughening benefits derived from M-A decomposition and matrix softening, limiting the further improvement of impact energy at the core compared to the 530 °C condition.

In summary, tempering at 580 °C followed by water quenching successfully restored overall section toughness by eliminating the key brittle M-A constituents. Nevertheless, the core remains the toughness weak link, limited by its coarse inherited microstructure and the evolution of unfavorable carbides. Furthermore, the water quenching step after tempering effectively suppressed impurity element (e.g., P, S) segregation at grain boundaries, thereby preventing reversible temper embrittlement. This ensured that the observed toughness improvement is solely attributable to beneficial microstructural evolution.

### 4.3. Anti-Softening Mechanism of Nanoscale Precipitates

While 580 °C tempering drastically improved toughness, such high-temperature exposure typically induces pronounced matrix recovery and softening, leading to strength loss [38]. Indeed, microstructural analysis revealed blurred lath boundaries and reduced dislocation density in the tempered state compared to the DQ condition. Despite this expected matrix softening, the steel retained high yield strength alongside high toughness. This macroscopic retention of strength suggests that a potent anti-softening mechanism is active, compensating for the dislocation recovery.

**Figure 10:**
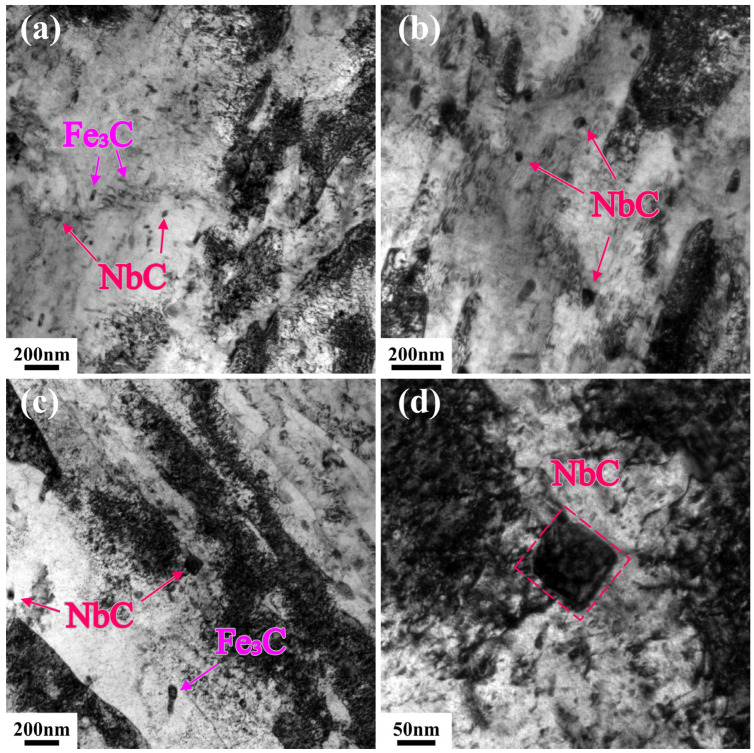
TEM morphology and distribution of nanoscale (Nb,Ti,Mo)C precipitates in the specimen tempered at 580 °C. (**a**,**b**) 0t; (**c**,**d**) 1/2t.

TEM examination (Figure 10) qualitatively reveals a significant population of fine, dispersed precipitates in the 580 °C tempered matrix. These square or near-spherical particles, 10–50 nm in size, are enriched in Nb, Ti, and Mo, identifying them as (Nb,Ti,Mo)C complex carbides. Unlike cementite or less stable VC, Nb-rich carbides possess exceptional thermal stability and low solubility in ferrite [39,40]. They resist Ostwald ripening even at 580 °C, maintaining their fine size and high number density.

This stable nanoprecipitation system is inferred to compensate for matrix softening via two complementary mechanisms. The primary mechanism is precipitation strengthening. Although a quantitative estimation was not performed due to the lack of statistical data on particle spacing, the ubiquitous presence of these fine, incoherent hard particles indicates that the Orowan bypassing mechanism plays a substantial role. As incoherent hard particles, they cannot be sheared by dislocations, compelling dislocations to bow around them. Fundamentally, the effective restriction of glissile dislocation motion by sessile obstacles serves as a critical governing factor for sustaining high flow stress [41]. In the present study, these thermally stable nanoprecipitates act as such effective immobile barriers. They pin mobile dislocations and generate significant back-stress, thereby effectively counteracting the softening effects of matrix recovery. Secondly, these nanoparticles inhibit matrix recovery by pinning lath interfaces and subgrain boundaries. This hinders the coalescence and growth of subgrains, thereby preserving a refined microstructure and retaining some grain boundary strengthening contribution.

In summary, the thermally stable (Nb,Ti,Mo)C nanoprecipitates, enabled by high-Nb microalloying, effectively counteract the strength loss from matrix recovery. They achieve this through a potent combination of direct precipitation strengthening and indirect microstructural stabilization by inhibiting recovery. This dual mechanism is the key to achieving an excellent strength–toughness balance after high-temperature tempering.

## 5. Conclusions

This study systematically investigated the through-thickness microstructural evolution and mechanical property homogeneity of a high-Nb microalloyed Q690 steel plate processed by direct quenching and tempering. The main conclusions are summarized as follows:

1. The AQ plate exhibited distinct microstructural heterogeneity, transitioning from a mixture of fine lath martensite and bainite at the surface to coarse granular bainite containing blocky M-A constituents at the core. High-temperature tempering at 580 °C provided sufficient thermodynamic driving force for the complete decomposition of the metastable M-A constituents into a ferrite matrix with finely dispersed carbides. This transformation effectively eliminated the primary initiation sites for brittle cracking, serving as the fundamental mechanism for the overall toughness improvement.
2. Significant homogenization was achieved for strength and hardness. Specifically, high-temperature tempering at 580 °C effectively eliminated the gradients within the plate interior, resulting in remarkable consistency between the quarter-thickness and the core region (e.g., Yield Strength: 744 MPa vs. 743 MPa; Hardness: 295 HV vs. 294 HV). However, the surface layer retained slightly higher strength and hardness, attributed to the hereditary structural refinement of the matrix from the rapid quenching stage.
3. In contrast to the uniform strength distribution, impact toughness still exhibited a noticeable through-thickness gradient. Although the core toughness was dramatically improved (to ~49 J) due to M-A decomposition, it remained lower than that of the surface and quarter-thickness layers (>120 J). This persistent gradient is attributed to the coarse granular bainitic matrix and the grain boundary carbide networks inherited from the core’s initial microstructure, which could not be fully refined during tempering.
4. The high yield strength level was successfully retained after 580 °C tempering due to a balance between strong precipitation strengthening from thermally stable (Nb,Ti,Mo)C nanoprecipitates and matrix recovery softening. To further enhance through-thickness homogeneity, future efforts should focus on disrupting the coarse inherited microstructure at the core, potentially through compositional adjustments to refine the as-quenched grain size or advanced thermomechanical processing to optimize carbide distribution.

## Figures and Tables

**Table 1:** Nominal and actual chemical compositions of the experimental steel (wt.%).

Element	C	Si	Mn	P	S	Cr	Mo	Nb	Ti	Al	B	Fe
Nominal	0.15	0.25	1.45	-	-	0.40	0.18	0.04	0.015	0.025	0.0015	Bal.
Actual	0.153	0.26	1.46	0.012	0.003	0.41	0.18	0.04	0.016	0.028	0.0014	Bal.

Note: The hyphen (-) indicates that the element is not specified in the nominal design.

## Data Availability

The original contributions presented in this study are included in the article. Further inquiries can be directed to the corresponding authors.

## References

[1] Gu L., Liu J., Lu S., Zhang S., Liu C. (2018). Research and Development of the Advanced High Strength Steel for the Mining Machinery. IOP Conf. Ser. Mater. Sci. Eng..

[2] Aftandiliants Y., Gnyloskurenko S. Development of Advanced Alloy Steels for Machinery. Proceedings of the 20th International Scientific Conference “Engineering for Rural Development”.

[3] Ohaeri E.G., Szpunar J.A. (2022). An Overview on Pipeline Steel Development for Cold Climate Applications. J. Pipeline Sci. Eng..

[4] Liu Y., Wu H., Ma H., Chen X., Huo X., Du L. (2025). Role of V and N Addition on the Microstructure and Mechanical Properties of Q690 MPa Medium and Heavy Plates Produced by Thermomechanically Controlled Processing. Steel Res. Int..

[5] Wang Q., Ye Q., Tian Y., Fu T., Wang Z. (2021). Superior Through-thickness Homogeneity of Microstructure and Mechanical Properties of Ultraheavy Steel Plate by Advanced Casting and Quenching Technologies. Steel Res. Int..

[6] Wang Q., Ye Q., Wang Z., Kan L., Wang H. (2020). Thickness Effect on Microstructure, Strength, and Toughness of a Quenched and Tempered 178 Mm Thickness Steel Plate. Metals.

[7] Zheng K., Zhang L., Hu C., Hu L., Wu K. (2025). Effect of Cross-Section on Low-Temperature Fracture Toughness of Marine Engineering Steel Thick Plate. Materials.

[8] Zhang X., Li G., Zhao H., Gao J., Wu H., Zhang C., Huang Y., Wu G., Wang S., Mao X. (2024). Evolution of Microstructure and Mechanical Properties along the Thickness Direction of 500 MPa HSLA Steel Heavy Plates. Mater. Sci. Eng. A.

[9] Krbata M., Ciger R., Kohutiar M., Eckert M., Barenyi I., Trembach B., Dubec A., Escherova J., Gavalec M., Beronská N. (2023). Microstructural Changes and Determination of a Continuous Cooling Transformation (CCT) Diagram Using Dilatometric Analysis of M398 High-Alloy Tool Steel Produced by Microclean Powder Metallurgy. Materials.

[10] Saastamoinen A., Kaijalainen A., Nyo T.T., Suikkanen P., Porter D., Kömi J. (2019). Direct-Quenched and Tempered Low-C High-Strength Structural Steel: The Role of Chemical Composition on Microstructure and Mechanical Properties. Mater. Sci. Eng. A.

[11] Hannula J., Kaijalainen A., Porter D.A., Somani M.C., Kömi J. (2021). Evaluation of Mechanical Properties and Microstructures of Direct-quenched and Direct-quenched and Tempered Microalloyed Ultrahigh-strength Steels. Steel Res. Int..

[12] Zhou N., Chai F., Luo X., Wang W., Gao F. (2024). The Effect of Direct Quenching on the Microstructure and Mechanical Properties of NiCrMo and Cu-Bearing High-Strength Steels. Materials.

[13] Shi J., Yu W., Yang X., Wang Y. (2024). Effect of Tempering Temperature on the Microstructure and Mechanical Properties of an Online Relaxation and Quenching High-Strength Steel. J. Mater. Eng. Perform..

[14] Makeshkumar M., Anburaj J., Kumar M.S., Santhosh A.J. (2023). Effect of Zirconium and Niobium on the Microstructure and Mechanical Properties of High-Strength Low-Alloy Cast Steels. Mater. Res. Express.

[15] Bai S., Li D., Li L., Lu H., Xing J., Bai P., Li J., Yan Z., Huang Z. (2024). Realizing the Strength-Ductility Balance of a Warm-Rolled 10 Mn Steel via Preparing Dual Nano-Sized Precipitates. J. Mater. Res. Technol..

[16] Liu G., Li Y., Liao T., Wang S., Lv B., Guo H., Huang Y., Yong Q., Mao X. (2023). Revealing the Precipitation Kinetics and Strengthening Mechanisms of a 450 MPa Grade Nb-Bearing HSLA Steel. Mater. Sci. Eng. A.

[17] DiGiovanni C., Rampelberg C., Zhou T.T., Cathcart C., Amirkhiz B.S., Zurob H., Scott C. (2024). Impact of Boron and Titanium on the Mechanical Properties and Recrystallization Behaviour of Low Carbon Cold Rolled and Batch Annealed HSLA Steels. Mater. Sci. Eng. A.

[18] Wang S., Gao Z., Wu G., Mao X. (2022). Titanium Microalloying of Steel: A Review of Its Effects on Processing, Microstructure and Mechanical Properties. Int. J. Miner. Metall. Mater..

[19] Fu W., Li C., Di X., Fu K., Gao H., Fang C., Lou S., Wang D. (2022). Improvement of Cu-Rich Precipitation Strengthening for High-Strength Low Carbon Steel Strengthened via Ti-Microalloying. Mater. Lett..

[20] Chen W., Gao P., Wang S., Zhao X., Zhao Z. (2020). Strengthening Mechanisms of Nb and V Microalloying High Strength Hot-Stamped Steel. Mater. Sci. Eng. A.

[21] Zhang X., Sun C., Li R., Sun Z., Zhang Y., Zhao Y., Chen W., Wu Y., Zhao N., Liu L., Zhang L. (2024). Effect of Nb Content on Mechanical Properties and Microstructure of High Strength Low Alloy Steel. Proceedings of the 12th International Conference on Advanced Materials and Engineering Materials.

[22] Esterl R., Sonnleitner M., Gschöpf B., Schnitzer R. (2019). Influence of V and Nb Micro-alloying on Direct Quenched and Tempered Ultra-high Strength Steels. Steel Res. Int..

[23] Yu Q., Wang Z., Liu X., Wang G. (2004). Effect of Microcontent Nb in Solution on the Strength of Low Carbon Steels. Mater. Sci. Eng. A.

[24] Zhao W., Zhou H., Fang L., Bai F., Yi H., Ali N., Zhang L., Zhen G. (2021). Study on Diversified Carbide Precipitation in High-strength Low-alloy Steel during Tempering. Steel Res. Int..

[25] Liang G., Mudawar I. (2017). Review of Spray Cooling—Part 2: High Temperature Boiling Regimes and Quenching Applications. Int. J. Heat Mass Transf..

[26] Yang C., Xu T., Zhao H., Hu C., Dong H. (2023). Regulation Law of Tempering Cooling Rate on Toughness of Medium-Carbon Medium-Alloy Steel. Materials.

[27] Liu Y., Ma H., Wang Z., Chen X., Huo X., Wu H., Du L. (2025). Effect of Heat Input on Microstructural Evolution and Impact Toughness of the Simulated CGHAZ for a Novel Q690 MPa V-N Medium and Heavy Plate. Materials.

[28] Zhang Z., Sun X., Wang Z., Li Z., Yong Q., Wang G. (2015). Carbide Precipitation in Austenite of Nb–Mo-Bearing Low-Carbon Steel during Stress Relaxation. Mater. Lett..

[29] Gladman T. (1999). Precipitation Hardening in Metals. Mater. Sci. Technol..

[30] Pan Z., Wang E., Wu H. (2024). Precipitation Behavior and Strengthening–Toughening Mechanism of Nb Micro-Alloyed Direct-Quenched and Tempered 1000 MPa Grade High-Strength Hydropower Steel. Metals.

[31] Jo H.-H., Kim K.-W., Park H., Moon J., Kim Y.-W., Shim H.-B., Lee C.-H. (2023). Estimation of Cooling Rate of High-Strength Thick Plate Steel during Water Quenching Based on a Dilatometric Experiment. Materials.

[32] Krbata M., Krizan D., Eckert M., Kaar S., Dubec A., Ciger R. (2022). Austenite Decomposition of a Lean Medium Mn Steel Suitable for Quenching and Partitioning Process: Comparison of CCT and DCCT Diagram and Their Microstructural Changes. Materials.

[33] Li Z., Tian L., Jia B., Li S. (2015). A New Method to Study the Effect of M–a Constituent on Impact Toughness of IC HAZ in Q690 Steel. J. Mater. Res..

[34] Ramachandran D.C., Moon J., Lee C.-H., Kim S.-D., Chung J.-H., Biro E., Park Y.-D. (2021). Role of Bainitic Microstructures with M-a Constituent on the Toughness of an HSLA Steel for Seismic Resistant Structural Applications. Mater. Sci. Eng. A.

[35] Wang J., Van Der Wolk P.J., Van Der Zwaag S. (2000). On the Influence of Alloying Elements on the Bainite Reaction in Low Alloy Steels during Continuous Cooling. J. Mater. Sci..

[36] Xia T., Ma Y., Zhang Y., Li J., Xu H. (2024). Effect of Mo and Cr on the Microstructure and Properties of Low-Alloy Wear-Resistant Steels. Materials.

[37] Mohseni P., Solberg J.K., Karlsen M., Akselsen O.M., Østby E. (2014). Cleavage Fracture Initiation at M–a Constituents in Intercritically Coarse-Grained Heat-Affected Zone of a HSLA Steel. Met. Mater. Trans. A.

[38] Xu W., Xie L., Liu X., Wang J., Xu Y., He M., Hu K., Liu C., Yu W. (2024). The Fabrication of Ultrahigh-Strength Steel with a Nanolath Structure via Quenching–Partitioning–Tempering. Materials.

[39] An X., Cao W., Zhang X., Yu J. (2024). Suppress Austenite Grain Coarsening by Nb Alloying in High–Temperature–Pseudo–Carburized Bearing Steel. Materials.

[40] Lei X.-W., Yang R.-B., Liu J.-M., Zeng L.-F., Lai C.-B., Luo X. (2021). Solubility Product and Equilibrium Equations of Nonstoichiometric Niobium Carbonitride in Steels: Thermodynamic Calculations. Met. Mater. Trans. A.

[41] Banerjee T., Sharma A., Picak S., Lattemann M., Singh P. (2025). An Atomistic Study Connecting Underlying Dislocation Behavior with Superior Mechanical Properties of NiCoCr Medium Entropy Alloy. Mater. Today Commun..

